# Life-course approach for maternal and child health: much to do

**DOI:** 10.1016/j.lansea.2023.100263

**Published:** 2023-08-04

**Authors:** The Lancet Regional Health – Southeast Asia

The WHO South-East Asia Region (SEAR) accounts for nearly a quarter of the world's population, with nearly 170 million individuals under 5 years of age and almost 35 million births in 2020. South Asia accounted for 25% of the babies born worldwide in 2020, with India recording almost 25 million births every year. However, this region also bears a heavy burden of maternal and child deaths. SEAR reported a maternal mortality ratio (MMR) of 152 deaths per 100,000 live births, an under-5 mortality rate of 32 deaths per 1000 live births, and a neonatal mortality rate (NMR) of 20 deaths per 1000 live births in 2019. A large number of these maternal and child deaths remain preventable but account for a large fraction of the global burden. Nearly one fifth of all maternal deaths (18%; 53,000 maternal deaths, as of 2017) and around one fifth of under-5 deaths (21%, 1.1 million, as of 2019) occur in SEAR. While the Sustainable Development Goals (SDGs) have set specific targets for countries to reduce their NMR to below 12 deaths per 1000 live births, under-5 mortality rate to below 25 deaths per 1000 live births, and MMR to below 70 deaths per 100,000 live births by 2030, recent stagnation in maternal and newborn health outcomes poses challenges in achieving these targets.

Broad social determinants like poverty often lead to under nutrition among women, while inadequate water, sanitation, and hygiene conditions make them susceptible to infections. Maternal education plays a crucial role in determining access to antenatal care and health services. Additionally, cultural beliefs, social support, and dietary patterns impact health-related behaviours and health-care use during pregnancy, affecting both maternal and neonatal outcomes. A study by Hettiarachchi and colleagues, in this issue, uncovers the burden of heart disease complicating pregnancy in Sri Lanka using a national Maternal Death Surveillance Response system. These deaths are largely preventable, but only if the broad social determinants are addressed. The authors recommend that preventing maternal mortality from heart disease in low-income and middle-income countries requires a lifecycle approach with situation-specific interventions and highly specialised care. Community awareness, capacity building related to management, and specific infrastructure development will be key strategies. Strengthening data systems in various countries is also necessary to monitor and improve maternal, newborn, and child health programmes effectively.

Within SEAR, about 52% of under-5 mortality is attributed to neonatal deaths. The leading causes of under-5 mortality are complications of prematurity, followed by pneumonia and diarrhoea, while the most common causes of neonatal mortality are prematurity, birth asphyxia, and neonatal infections. To prevent maternal and child mortality, a key focus should be on addressing women's vulnerabilities during the preconception period and pregnancy. These vulnerabilities not only impact a woman's own health but are also transferred to her offspring, leading to increased risks of preterm birth and foetal growth restriction. The implementation of interventions to ensure positive pregnancy outcomes is crucial during antenatal care. However, addressing the social determinants that negatively affect pregnant women's health-seeking behaviour is equally vital. These determinants include root causes such as poverty, unsafe living environments, lack of education and agency, and limited access to quality antenatal care and health services. In this issue, Memon and colleagues reveal within-district geospatial clustering of under-5 mortality in Pakistan, associated with social factors, poor community health worker coverage, and distance from health facilities, underscoring the disparities in child health.

Despite substantial progress in reducing child and maternal mortality (62% reduction in NMR and 57% in MMR) over the past two decades, the current rates of progress are insufficient to achieve the SDGs. Quality of care remains a major problem: the *Lancet Global Health* Commission on High-Quality Health Systems estimated that 66% of all deaths amenable to medical care in India occur because of poor quality as opposed to non-utilisation. For example, in Uttar Pradesh, India, facility-based birth attendants did only 40% of items on the WHO safe childbirth checklist in a typical birth. While coverage rates have increased to 75% in recent years, effective coverage remains low, leading to deliveries in facilities without the capacity to handle maternal and newborn complications adequately. Kruk and colleagues discuss in this issue that guiding women to health facilities lacking life-saving services based on a determination of “low risk” may undermine efforts to reduce maternal and newborn mortality in India.

The recently published *Lancet* Series on Small Vulnerable Newborns proposed a new definition and conceptual framework, categorising preterm birth, small for gestational age, and low birth weight under the term “small vulnerable newborns” (SVN) in an attempt to enhance problem definition and programming for SVN prevention. The Commission recommends implementing eight proven interventions aimed at SVN prevention that would result in a healthier start for live-born infants, while also reducing the number of stillbirths, improving maternal health, and contributing to a positive economic and social development in society.

Tackling maternal and child mortality in SEAR requires effective antenatal care and comprehensive health care across the life course to achieve positive outcomes. Taking actions now is paramount to create a thriving society where women and children flourish, resulting in healthier individuals, families, and societies. By adopting a data-driven and critical approach, we can strive towards reducing the prevalence of maternal and child mortality and achieving the SDGs.

